# Perceived Decline in Straight Blade Direct Laryngoscopy Skills in the Era of Video Laryngoscopy: An Exploratory Pilot Survey Study

**DOI:** 10.7759/cureus.93089

**Published:** 2025-09-24

**Authors:** Lawrence W Chinn, Myriam Lin, Dhvani Shihora

**Affiliations:** 1 Anesthesiology and Perioperative Medicine, Rutgers University New Jersey Medical School, Newark, USA

**Keywords:** airway education, airway training, anesthesiology training, direct laryngoscopy, resident education, skill erosion, video laryngoscopy

## Abstract

Background

Video laryngoscopy is increasingly used for airway management and is often chosen for both routine and complex intubations. While video laryngoscopy offers clear advantages, direct laryngoscopy remains an important fallback technique when visualization is impaired or equipment fails. As video laryngoscopy becomes more common, questions have been raised about whether reliance on this technology may reduce opportunities for trainees to practice and maintain direct laryngoscopy skills.

Methods

We conducted a cross-sectional survey of anesthesiology residents and attending anesthesiologists at a single academic institution. The survey assessed self-reported confidence using the Miller blade, frequency of use, perceived importance of maintaining this skill, and beliefs about the effectiveness of current teaching practices. Responses were recorded using a 1-5 Likert scale. Descriptive statistics and Spearman correlation coefficients were calculated.

Results

Twenty-three clinicians completed the survey (12 residents, 11 attendings). Residents reported low confidence (mean 2.5) and infrequent use (mean 2.2) of the Miller blade but rated its importance as relatively high (mean 3.75). Confidence correlated strongly with frequency of use (ρ=0.77, p=0.0031). Attendings reported limited teaching (mean 1.8), acknowledged the importance of maintaining direct laryngoscopy (DL) (mean 3.8), and expressed moderate agreement that these skills are at risk of becoming a "lost art" (mean 3.36).

Conclusions

This exploratory pilot study suggests that anesthesiology residents and attendings perceive direct laryngoscopy skills as important yet underemphasized in training. Given its small, single-center design, these findings are not generalizable but highlight the need for multi-center studies to further evaluate how reliance on video laryngoscopy may influence preservation of foundational airway techniques.

## Introduction

Video laryngoscopy (VL) has become a cornerstone of modern airway management. Initially introduced for difficult airways, VL is now routinely used across a range of clinical scenarios, from high-risk intubations to standard elective cases. Randomized trials demonstrate that VL improves glottic visualization and first-pass success, even in patients without predicted airway difficulty [1-6]. Additional studies confirm its advantages in patients with cervical spine immobilization, head and neck tumors, and other predictors of difficult intubation, with reduced laryngoscopy force and cervical spine motion compared to direct laryngoscopy (DL) [7]. These benefits have supported the broad adoption of VL across operating rooms, ICUs, and emergency settings.

Despite VL's advantages, DL remains an essential fallback technique. Airway guidelines from the American Society of Anesthesiologists and the Difficult Airway Society emphasize that clinicians must remain proficient in multiple techniques, including DL, to ensure readiness when visualization is obscured by blood, secretions, or device malfunction [8,9]. Straight blade laryngoscopy with the Miller blade retains clinical relevance in pediatric airways, patients with anterior laryngeal anatomy, and situations where camera visualization fails [10,11]. These scenarios underscore the ongoing importance of maintaining DL skills as a safety net.

As VL becomes increasingly dominant, hands-on exposure to DL may decline, particularly among trainees. Residents may graduate with limited DL experience, especially with straight blades like the Miller, potentially reducing confidence in fallback readiness. This concern is not unique to anesthesiology; procedural specialties such as surgery and endoscopy have documented "deskilling" effects when reliance on robotics or AI-assisted technology displaces foundational manual practice [12-14]. A similar dynamic may exist in airway management. While the Miller blade remains essential in scenarios such as pediatric intubation or when secretions obscure the VL view, it may now be encountered only rarely in training, not due to disinterest, but due to decreased opportunity.

Importantly, this study does not seek to demonstrate actual skill decay, nor to assert that video laryngoscopy should be curtailed. Rather, it aims to explore clinician perceptions of confidence, usage, and teaching of foundational techniques in a VL-driven landscape. Perceived gaps in training are educationally relevant, even when they reflect reduced exposure rather than actual incompetence. By quantifying these perceptions, we can begin to understand how reliance on advanced tools may quietly displace deliberate practice-and how that shift is being felt by clinicians at different stages of training.

This exploratory pilot survey study examined anesthesiology residents' and attendings' perceptions of Miller blade DL training in a VL-dominant era. Specifically, we assessed confidence, frequency of use, perceived importance, and beliefs about how teaching has changed. While this single-site study is not intended to draw generalizable conclusions, it offers early data to inform future research and training strategies aimed at preserving critical fallback skills when technology fails or is unavailable.

## Materials and methods

This cross-sectional survey was administered via REDCap to anesthesiology residents and attending anesthesiologists at a single academic medical center. Residents were asked to report their confidence in using the Miller blade for DL, frequency of use, perceived importance of maintaining this skill, and beliefs regarding whether foundational manual airway techniques are being adequately taught. Attendings were asked about the frequency with which they teach DL, their perceived importance of preserving it, and their agreement with the statement that DL is becoming a "lost art".

Survey items were developed de novo by the study team, as no validated instrument addressed this specific topic. Questions were based on expert consensus and clinical experience, and no formal pilot testing or psychometric validation was performed prior to distribution. The full survey instrument is provided in Appendix A.

While the survey focused specifically on the Miller blade, we acknowledge that the Macintosh blade remains more commonly used in routine clinical DL. The Miller blade was selected for its pedagogical significance in teaching fallback techniques.

Survey responses used ordinal scales (e.g., 1-4 for confidence, 1-5 for importance), and results were summarized using medians and interquartile ranges. Because the data were ordinal and not normally distributed, Spearman's rank correlation coefficients were calculated to assess associations between confidence, frequency of use, perceived importance, and beliefs about declining teaching among residents. Confidence scores were not assessed among attendings; therefore, no direct statistical comparisons were made between cohorts.

This study was approved by the Rutgers Institutional Review Board (IRB# Pro2025001199).

## Results

A total of 23 clinicians completed the survey, including 12 anesthesiology residents and 11 attending anesthesiologists. The overall response rate was 46%, with 33% of residents (12 of 36) and 65% of attendings (11 of 17) participating.

Among residents, the mean confidence score for performing Miller blade DL was 2.5 (on a 1-4 scale), and the mean frequency of use score was 2.2 (on a 1-5 scale). Residents rated the importance of maintaining Miller blade skills as relatively high (mean 3.75), and reported a high level of agreement with the belief that teaching of these skills is declining (mean 4.08).

**Figure 1:**
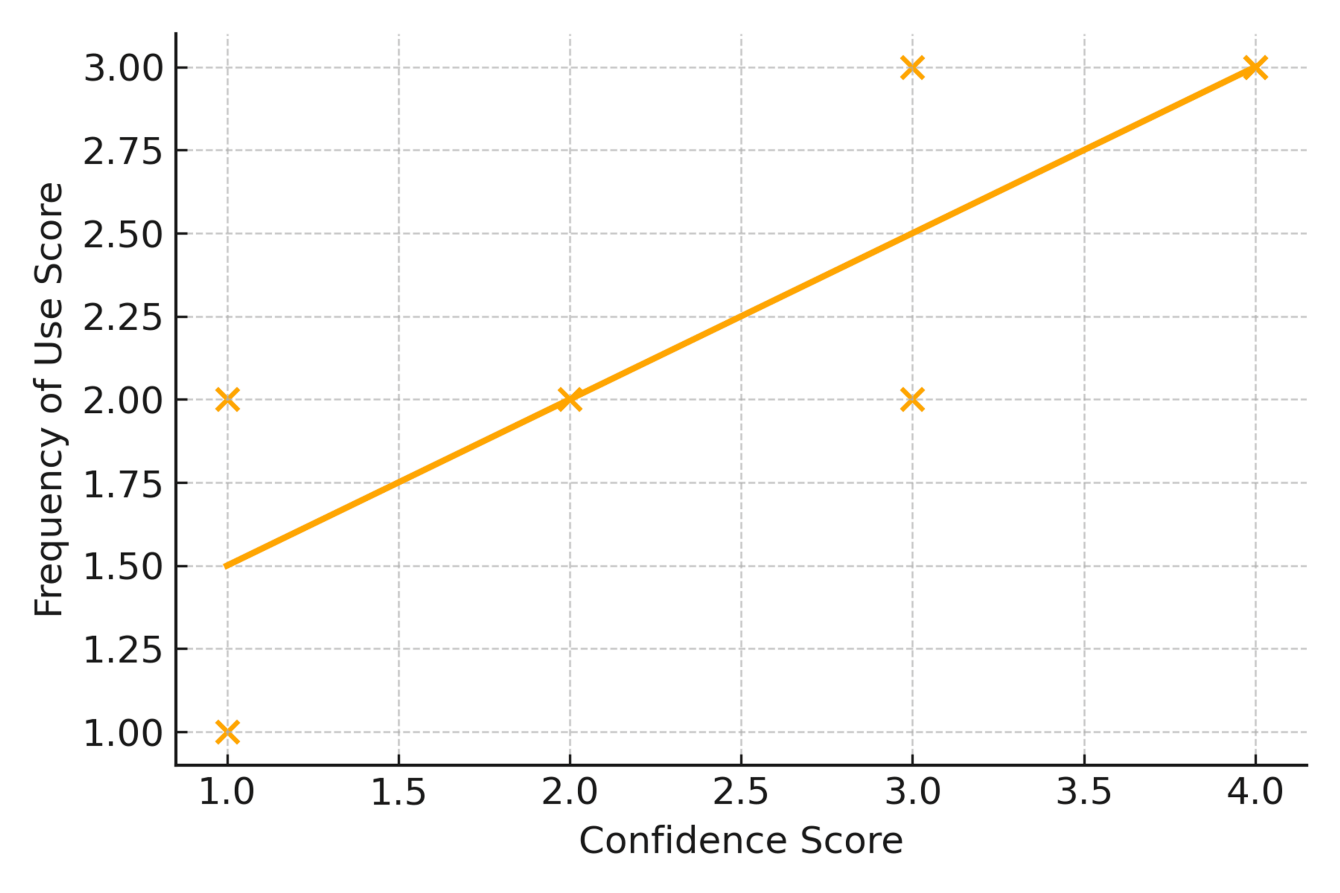
Residents' confidence versus frequency of Miller blade use Scatterplot with trend line showing the correlation between anesthesiology residents' self-reported confidence in using the Miller blade for direct laryngoscopy and their reported frequency of use. Each point represents one resident respondent. A positive association was observed (Spearman's ρ=0.77, p=0.0031).

Spearman correlation analyses among residents revealed a strong positive association between confidence and frequency of use (ρ=0.77, p=0.0031), and a moderate positive association between confidence and perceived importance (ρ=0.65, p=0.02). The correlation between confidence and the belief that DL teaching is declining was weak and not statistically significant (ρ=0.32, p>0.1). These relationships are illustrated in Figure 1.

Attendings reported a mean score of 1.8 for frequency of teaching DL techniques, a mean importance score of 3.8 for preserving these skills, and a mean agreement score of 3.36 with the statement that DL is becoming a "lost art". Confidence scores were not collected for attendings, and no direct statistical comparisons were made between resident and attending groups due to differences in survey structure.

## Discussion

In this exploratory pilot survey, anesthesiology residents reported low confidence and infrequent use of the Miller blade for DL, yet consistently rated the skill as important. Confidence was strongly correlated with frequency of use, underscoring the role of regular exposure in perceived competence. Attendings likewise endorsed the importance of preserving DL but reported only occasional teaching of this technique. Together, these findings suggest a potential gap between the acknowledged value of DL and the opportunities provided to practice and teach it.

The growing dominance of VL in airway management is supported by randomized trials demonstrating improved first-pass success and glottic visualization [1-7]. However, guidelines continue to emphasize that fallback strategies-including DL-must remain part of clinician training and preparedness [8,9]. Our findings support this concern from an educational standpoint: residents perceive DL as important but report limited practice opportunities and teaching exposure. These perceived gaps are educationally meaningful, even if not indicative of skill decay per se.

Comparable patterns have been observed in other procedural specialties. In surgical education, increasing reliance on robotic platforms has prompted discussion about declining competence in open surgical techniques [12,13]. In gastrointestinal endoscopy, Sinagra et al. described how reliance on artificial intelligence may reduce trainee autonomy and tactile judgment, raising concerns about procedural deskilling [14]. These parallels suggest that technology-driven displacement of manual practice is a broader educational issue, with implications for airway training in anesthesiology.

While we do not claim that DL skills are being lost outright, our findings suggest that reduced exposure and hands-on practice may be contributing to declining confidence. This is not a failure of residents or faculty, but rather a training environment that increasingly favors newer tools. Deliberate reinforcement of fallback techniques, such as DL with the Miller blade, may help maintain readiness when VL fails or is unavailable. Structured simulation, supervised repetition, and case-based review could help preserve these skills, as has been recommended in surgical and endoscopy training [12-14].

This study has several limitations. It was conducted at a single academic center with a modest sample size, limiting generalizability. The survey instrument was developed de novo and has not been formally validated. Confidence was self-reported and may not reflect actual clinical performance. Additionally, the study focused on the Miller blade specifically, which may not represent all forms of direct laryngoscopy. No comparisons were made between residents and attendings due to differences in survey design.

Despite these limitations, this pilot offers structured data on how clinicians perceive foundational skill preservation in a VL-dominant training environment. Future research should expand to multi-center designs, use validated instruments, and incorporate objective performance metrics to better understand how modern airway training impacts fallback technique readiness.

## Conclusions

VL has significantly advanced airway management and improved first-pass success, but it does not eliminate the need for DL. In this exploratory pilot survey, anesthesiology residents and attendings acknowledged the enduring importance of Miller blade skills while reporting limited confidence, infrequent use, and reduced teaching exposure. These findings suggest a potential training gap in preserving fallback techniques within a VL-dominant environment.

The study's single-center design, modest response rate, and use of a non-validated survey instrument limit generalizability. Nevertheless, the consistency of responses across both trainees and faculty highlights a perceived shift in training priorities that warrants further investigation. Multi-center studies using validated tools and objective skill assessments are needed to determine whether reliance on VL is contributing to reduced exposure and proficiency in foundational direct laryngoscopy techniques.

## Appendices

Appendix A

Resident Survey Instrument 

This survey was administered anonymously to anesthesiology residents.

Q1. What is your current postgraduate year (PGY/CA)?

PGY-1

PGY-2

CA-1

CA-2

CA-3

Q2. What type of residency program are you in?

Academic

Community

Other

Q3. Have you received formal training in using the Miller blade for intubation?

Yes

No

Unsure

Q4. How confident are you in your ability to use the Miller blade?

Not confident at all

Slightly confident

Moderately confident

Very confident

Extremely confident

Q5. How often have you used the Miller blade in clinical practice?

Never

Rarely

Sometimes

Often

Always

Q6. Do you believe that the Miller blade remains an important skill for anesthesiologists to learn?

Strongly disagree

Disagree

Neutral

Agree

Strongly agree

Q7. Have you noticed a decline in teaching of traditional airway techniques (e.g., Miller blade, lightwand) during your training?

Strongly disagree

Disagree

Neutral

Agree

Strongly agree

Appendix B

Attending survey instrument

This survey was administered anonymously to attending anesthesiologists.

Q1. What is your current age group?

Under 35

35-44

45-54

55-64

65+

Q2. How many years have you been practicing anesthesiology?

Less than 5

5-9

10-19

20+

Q3. Do you actively teach the use of the Miller blade to residents?

Yes

No

Sometimes

Q4. How frequently do you teach traditional airway techniques compared to video laryngoscopy?

Never

Rarely

Sometimes

Often

Always

Q5. Do you believe traditional airway management skills are becoming a lost art?

Strongly disagree

Disagree

Neutral

Agree

Strongly agree

Appendix C

Expanded survey results

**Table 1:** Resident Survey Responses (n = 12) This table summarizes responses from anesthesiology residents (n = 12) regarding their postgraduate year of training (PGY/CA), program type, formal training in the use of the Miller blade, and perceptions of confidence, frequency of use, importance, and teaching of direct laryngoscopy (DL) in the era of video laryngoscopy (VL). Items were coded numerically for analysis, with detailed category definitions provided in the last column. PGY/CA values reflect resident training levels (CA-1 through CA-3). Confidence, frequency of use, importance, and perceived decline in teaching were measured using 5-point Likert-type scales, where higher values indicate greater confidence, more frequent use, stronger perceived importance, or stronger agreement. Descriptive statistics are presented as mean, standard deviation (SD), median, minimum (Min), and maximum (Max). These data provide insight into residents’ self-reported experience with and perceptions of Miller blade DL skills.

Survey Item	Mean	SD	Median	Min	Max	Scale
PGY/CA	4.0	0.43	4	3	5	1 = PGY 1, 2 = PGY 2, 3 = CA-1; 4 = CA-2; 5 = CA-3
Program type	1.0	0.0	1	1	1	1 = Academic
Formal training (Miller blade)	1.25	0.45	1	1	2	1 = Yes; 2 = No; 3 = Unsure
Confidence in Miller blade	2.5	1.0	2.5	1	4	1 = Not at all confident; 2 = Slightly confident; 3 = Moderately confident; 4 = Very confident; 5 = Extremely confident
Frequency of use	2.17	0.58	2	1	3	1 = Never; 2 = Rarely; 3 = Sometimes; 4 = Often; 5 = Always
Importance of Miller blade	3.75	1.06	4	2	5	1 = Strongly disagree; 2 = Disagree; 3 = Neutral; 4 = Agree; 5 = Strongly agree
Perceived decline in teaching	4.08	0.90	4	2	5	1 = Strongly disagree; 2 = Disagree; 3 = Neutral; 4 = Agree; 5 = Strongly agree

**Table 2:** Attending Survey Responses (n = 11–12) This table presents responses from attending anesthesiologists (n = 11) regarding their experience, teaching practices, and perceptions of direct laryngoscopy (DL) in the era of video laryngoscopy (VL). Items were measured using Likert-type scales or categorical codes, with detailed anchors provided in the last column. Age group and years in practice are reported as ordinal categories. Teaching frequency and attitudinal questions (e.g., perception of DL as a “lost art”) were scored on 1–5 scales, where higher values indicate greater frequency or stronger agreement. Descriptive statistics are provided as mean, standard deviation (SD), median, minimum (Min), and maximum (Max). These results summarize how attendings view the preservation and teaching of Miller blade DL skills within current training environments.

Survey Item	Mean	SD	Median	Min	Max	Scale
Age group	3.33	1.23	3	1	5	1 = Age <35 years; 2 = 35–44 years; 3 = 45–54 years; 4 = 55–64 years; 5 = ≥65 years
Years in practice	3.17	0.94	3	1	4	1 = <5 years; 2 = 5–9 years; 3 = 10–19 years; 4 = ≥20 years
Teach Miller blade	1.82	0.87	2	1	3	1 = Yes; 2 = No; 3 = Sometimes
Frequency teaching DL vs VL	3.83	0.83	4	2	5	1 = Never; 2 = Rarely; 3 = Sometimes; 4 = Often; 5 = Always
Belief airway skills “lost art”	3.36	1.21	3	2	5	1 = Strongly disagree; 2 = Disagree; 3 = Neutral; 4 = Agree; 5 = Strongly agree
